# Health care seeking in modern urban LMIC settings: evidence from Lusaka, Zambia

**DOI:** 10.1186/s12889-022-13549-3

**Published:** 2022-06-16

**Authors:** Emma Clarke-Deelder, Doris Osei Afriyie, Mweene Nseluke, Felix Masiye, Günther Fink

**Affiliations:** 1 Department of Epidemiology and Public Health, Swiss Tropical & Public Health Institute, Basel, Switzerland; 2 University of Basel, Basel, Switzerland; 3 Directorate of Clinical Care and Diagnostic Services, Ministry of Health, Lusaka, Zambia; 4 Department of Economics, University of Zambia, Lusaka, Zambia

**Keywords:** Child health, Zambia, Primary care, Bypassing

## Abstract

**Background:**

In an effort to improve population health, many low- and middle-income countries (LMICs) have expanded access to public primary care facilities and removed user fees for services in these facilities. However, a growing literature suggests that many patients bypass nearby primary care facilities to seek care at more distant or higher-level facilities. Patients in urban areas, a growing segment of the population in LMICs, generally have more options for where to seek care than patients in rural areas. However, evidence on care-seeking trajectories and bypassing patterns in urban areas remains relatively scarce.

**Methods:**

We obtained a complete list of public health facilities and interviewed randomly selected informal sector households across 31 urban areas in Lusaka District, Zambia. All households and facilities listed were geocoded, and care-seeking trajectories mapped across the entire urban area. We analyzed three types of bypassing: i) not using health centers or health posts for primary care; ii) seeking care outside of the residential neighborhood; iii) directly seeking care at teaching hospitals.

**Results:**

A total of 620 households were interviewed, linked to 88 health facilities. Among 571 adults who had recently sought non-emergency care, 65% sought care at a hospital. Among 141 children who recently sought care for diarrhea, cough, fever, or fast breathing, 34% sought care at a hospital. 71% of adults bypassed primary care facilities, 26% bypassed health centers and hospitals close to them for more distant facilities, and 8% directly sought care at a teaching hospital. Bypassing was also observed for 59% of children, who were more likely to seek care outside of the formal care sector, with 21% of children treated at drug shops or pharmacies.

**Conclusions:**

The results presented here strongly highlight the complexity of urban health systems. Most adult patients in Lusaka do not use public primary health facilities for non-emergency care, and heavily rely on pharmacies and drug shops for treatment of children. Major efforts will likely be needed if the government wants to instate health centers as the principal primary care access point in this setting.

**Supplementary Information:**

The online version contains supplementary material available at 10.1186/s12889-022-13549-3.

## Background

Despite significant improvements over the past 30 years, mortality rates in LMICs remain high: 4% of children in LMICs die before their 5^th^ birthday, and preventable mortality from both infectious and chronic conditions is significantly higher than in high-income countries [1, 2]. Many efforts to improve health outcomes in LMICs have focused on improving access to primary health care services through interventions such as the removal of user fees for services in public primary health facilities [3–8]. However, there is widespread evidence that the average quality of care provided in health facilities in many LMICs is low [9–16]. In addition, quality of care tends to vary significantly across health facilities, creating a complex decision-making environment for patients who seek care [17–19].

There is growing evidence that patients in LMICs are increasingly aware of differences in quality of care, and often bypass primary health facilities in their communities to seek care at more distant or higher-level health facilities [20]. Extensive bypassing has been documented for childbirth [21–28]: for example, in a study in Uganda, 29% of women bypassed their nearest health facility for delivery [25]; in a study in Nepal, 71% of women whose nearest facility was a birthing center bypassed the center to deliver in a hospital [24]. Studies have also documented high rates of bypassing for primary care in settings such as China, Ghana, India, and Chad [29–32], and for inpatient care in Sierra Leone and Kenya [33, 34]. Fewer studies have examined bypassing for pediatric care [34–36], but these studies also show high rates of bypassing. Important predictors of bypassing include distance to a hospital [28] and perceived quality of the local primary health facility [22, 32, 37]. Bypassing in urban areas, where patients have more options for where to seek care and their choices are less constrained by distance, may be particularly revealing of patient preferences. While evidence on bypassing patterns in urban areas is relatively scarce, the existing evidence suggests that there are often higher rates of hospital use in urban areas relative to rural areas [31, 35, 38].

In this study, we describe care-seeking patterns among urban informal sector households in Lusaka, Zambia. Thanks to a 2012 reform [6] patients in Lusaka are not required to pay fees for primary care as long as they access care through health posts or health centers. Despite these financial incentives to use lower level facilities, there is evidence that many families bypass local health centers and directly seek care either at hospitals or in the private sector [39].

To assess the extent of bypassing, we collected detailed treatment seeking data from 620 randomly-selected households in Lusaka, and identified the location and type of facilities used for adult as well as child healthcare. We quantify the rates of three types of bypassing: i) not using health centers or health posts for primary care (non-compliance with government recommendations); ii) seeking care outside of the residential neighborhood (spatial bypassing to reach higher quality facilities), and iii) directly seeking care at tertiary teaching hospitals (bypassing two levels of care).

## Methods

### Study setting

Zambia is a lower-middle-income country in southern Africa with a life expectancy at birth of 64 years, maternal mortality rate of 213 deaths per 100,000 live births, and child mortality ratio of 62 deaths per 1,000 live births [1]. In 2019, 44% of the population lived in an urban area [1]. Lusaka district, including the capital city, has a population of approximately two million people living in an area of approximately 418 square kilometers. In Lusaka province (of which 80% is Lusaka district), average household wealth, infrastructure, education levels, and access to health care services are generally higher than in other parts of Zambia. For example, in 2018, 50% of the population of Lusaka province was in the country’s highest wealth quintile; 98% had access to an improved source of drinking water compared with 71% nationwide; the female literacy rate was 80% compared with 66% nationwide; and 91% of live births in the preceding five years were in a health facility compared with 84% nationwide [39].

The Zambian health system has a pyramid-structure with three levels. Level 1 includes health posts (with catchment areas of 500 households in rural areas and1000 households in urban areas), health centers (with catchment areas of 10,000 in rural areas and 50,000 in urban areas), mini hospitals (catchment population between 50,000 and 80,000) and district hospitals (catchment population between 80,000 and 20,000). Level 2 includes provincial level hospitals (catchment population 200,000 to 800,000) which provide secondary care and curative care in pediatrics, obstetrics and gynecology and general surgery. Level 3 includes tertiary hospitals (catchment population 800,000 and above), such as the University Teaching Hospital in Lusaka, and specialized hospitals, such as the Cancer Diseases Hospital and the National Heart Hospital. Residential neighborhoods are generally assigned to a nearby health center or health post where they are expected to go as their first point-of-contact with the health system; they may then be referred to a hospital if needed. In practice, residents may choose to go to a different health center or health post from the one they are assigned to; in these cases, they do not incur a bypassing fee because they are still accessing the system at the recommended level. However, if they seek care directly at a hospital, then they incur a bypassing fee.

In addition to the public system, there are private and not-for-profit health facilities throughout Zambia. These are registered by the National Health Professions Council [40]. In Lusaka, these are mainly health centers and Level 1 hospitals.

At the data of data collection, residents of Lusaka mainly used Level 1 and Level 3 care, as there were few Level 2 hospitals in the city. Since data collection, many health facilities in Lusaka have been upgraded in levels. Throughout this paper, we focus on the levels as they were at the time of data collection.

### Study design

This study was a cross-sectional household survey conducted in Lusaka district in Zambia from November to December 2020.

### Study population and sample

The target population for the study was all adults employed in the informal sector and aged between 18–65 years who lived in Lusaka district, and their children. We define the informal sector as businesses or other economic units that are not registered with a tax or licensing authority. Those who are employed in the informal sector tend not to have contracts or entitlements. As of 2014, the informal sector accounted for about 90% of employment in Zambia [41]. To determine whether respondents were employed in the formal or informal sector, we asked whether they had a formal employment contract and contributed to the National Pension Scheme Authority (NAPSA).

We used a random clustered sampling approach to select households for participation in this study. The target sample size of 700 households was chosen for the purposes of a separate analysis of health insurance participation and health system confidence. To draw the sample, we first randomly sampled 35 enumeration areas (EAs) from the 1,225 listed in the 2010 Zambia Census of Population and Housing. Within each EA, we then approached every fourth household until we reached a sample of 20 informal sector households. Eligible heads of households or their spouse were provided information about the study and those who consented were interviewed using the questionnaire.

For the purposes of this analysis, we defined the adult analytic sample to include all adults whose most recent health visit was for care for a chronic condition, a check-up, or a new (acute) health issue. We excluded adults whose most recent health visit was an emergency. We defined the child sample to include all children aged five and under who had received care in the past two weeks for fever, diarrhea, cough, or fast breathing.

### Data collection

Interviewers were trained and supervised directly by a member of the study team (DOA). Household interviews were conducted from November 6 to December 19, 2020. During interviews, adults in the sample were asked about their own care-seeking during their most recent health visit, as well as care-seeking for fever, diarrhea, cough, or fast breathing in the past two weeks for children aged five and under in their household (up to a total of five children per household).

All data were collected using the Open Data Kit (ODK) software package on hand-held tablets. Survey tools were developed in English and then translated to local languages by the survey team. Interviews were conducted in the respondent’s preferred language (English, Nyanja, or Bemba). Residential coordinates for all households were collected directly through the tablets using a geolocation function integrated into ODK.

In addition, we collected information on the locations of health facilities in Lusaka. An initial list of facilities as well as their geolocations was obtained from the Zambian Ministry of Health. This list included public facilities as well as private and not-for-profit (e.g., religious) health facilities. It did not include pharmacies or drug shops. Geocodes of all facilities in the sample were verified by one of the authors (DOA) in January 2021 through a combination of online mapping resources (January 10–15) [42] and personal visits to facilities (January 17–22).

### Ethics

We obtained ethical clearance from the University of Zambia Social Sciences and Humanities Ethical Clearance Committee (HSSREC-2020-SEP-012) and authority to conduct research from the National Health Research Authority (NHRA00018/15/10/2020). We also obtained ethical clearance from the Ethikkommission Nordwest- und Zentralschweiz (EKNZ) in Switzerland (AO_2020-00,029).

### Primary outcome variables

The primary outcome was bypassing. We used three definitions of bypassing (Table 1). These definitions are not mutually exclusive, but each measure different bypassing constructs with different interpretations. First, we defined “primary care bypassing” as using a health facility other than a health center or health post for any non-emergency care. This strict definition of bypassing aligns with guidelines from Zambia’s Ministry of Health. Second, we defined “horizontal bypassing” as using a distant health facility or a pharmacy rather than a nearby facility for non-emergency care – this type of bypassing implies additional transport time and cost, and is likely a reflection of households anticipating to find higher quality of care outside of their residential areas. To identify nearby facilities, we asked all subjects in each neighborhood about the facility their neighborhood belonged to. In most cases, the large majority of respondents agreed on one specific facility. In some cases, two primary facilities were mentioned. We defined nearby facilities as the one (if only one was mentioned) or two (if two were mentioned) facilities that respondents mentioned, as well as the facility that was spatially closest to the respondent (if this was different from the one or two facilities mentioned). Of note, Ministry of Health guidelines do not specify which specific health facility people should go to for care, so horizontal bypassing can in principle be in line with Ministry of Health guidelines as long as people seek care for non-emergency conditions at a health centre or health post rather than a hospital. In practice, many patients seeking care outside of their residential area seek care at higher level facilities, in which case horizontal bypassing also implies primary care bypassing. Last, we defined “two-level” bypassing as using a teaching hospital (Level 3) for non-emergency care. Patients who do this are bypassing not only the available primary health care facilities but also the regular (Level 1, non-teaching) hospitals.

**Table 1:** Definitions of bypassing

Type of bypassing	Definition
Primary care bypassing	Using a facility other than a health centre or health post for non-emergency care
Horizontal bypassing	Using a distant facility rather than a nearby facility for non-emergency care; nearby facilities include those spatially closest as well as those listed by respondents as the main facility their neighborhood belonged to
Two-level bypassing	Using a teaching hospital (Level 3) for non-emergency care

### Statistical analysis

We began our analysis by describing the characteristics of the adult and child analytic samples. We described respondents’ demographic characteristics (e.g., gender and age) as well as the landscape of health facilities in the area the where respondents lived. To describe the landscape of health facilities, we calculated the number of health facilities within 1 km and within 5 km of where each respondent lived using Euclidean distance and then took the average across respondents.

Next, we mapped and described the spatial distribution of the health facilities in Lusaka and the types of facilities that adults and children in the study sample visited. Mapping included any facilities on the Ministry of Health’s list of health facilities, but it did not include pharmacies or drug shops, even though some respondents sought care in these locations.

We then calculated the rate of bypassing (using all three definitions above) for adults and children in the sample, disaggregated by the reason for their health visit. We mapped care-seeking patterns for each study participant meeting each of the three definitions of bypassing using QGIS Version 3 [43]. In addition, we examined how bypassing patterns varied across constituencies. Constituencies are administrative areas that contain multiple EAs; Lusaka has 7 constituencies covering 1,125 EAs.

Finally, we used logistic regression to analyze associations between study participant characteristics (including sex, age, marital status, education level, wealth measured using an asset score, and reason for seeking care) and each of the three types of bypassing. We fit models in the adult and child samples separately. We clustered standard errors at the EA level. All analyses were conducted using Stata 16 [44].

## Results

A total of 753 randomly selected households were approached by the study team. Nine households (1.2%) were excluded because the respondent was above 65, 43 households (5.7%) could not be reached and 26 (3.5%) indicated they were too busy or not interested in the study. Forty-eight households (6.4%) were employed in the formal sector, and also excluded from the study. We therefore interviewed 627 adults about their recent care-seeking behavior and that of children in their household. Three EAs had less than four eligible households due to high formal sector employment in these neighborhoods – we excluded households from these areas from the analysis (*N* = 7, 0.9%) because the number of observations was too small to establish the most commonly used health facilities in these settings. A sample flow diagram is included in Additional file 1: Figure S1.

The final adult analytic sample included the 577 adults whose most recent visit to a health facility was for non-emergent care. The majority (78%) of participants were female (Table 2). About one quarter (24%) of the sample was over age 45, 43% was aged 30–44, and 29% was under age 30. The majority (59%) of the sample had completed secondary education or higher. The most common reason for their most recent health visit were new health problems (54%), followed by routine check-up (24%), and chronic disease treatment (22%). On average, the households in the sample had two general hospitals, 16 private facilities, and 11 other health facilities within five kilometers of their homes.

**Table 2:** Descriptive statistics

	(1) Adult sample (*N* = 577)	(2) Child sample (*N* = 141)
*Demographic characteristics*	*N (%)*	*N (%)*
Female	447 (77.5%)	69 (48.9%)
Age under 30	165 (28.6%)	-
Age 30–44	250 (43.3%)	-
Age 45 plus	142 (24.6%)	-
Primary education or less	234 (40.6%)	-
Secondary education	256 (44.4%)	-
Higher education	87 (15.1%)	-
Married	394 (68.3%)	-
	*Mean (SD)*	*Mean (SD)*
Asset quintile	3.0 (1.4)	2.7 (1.2)
*Reason for seeking care*	*N (%)*	*N (%)*
Emergency visit	0 (0.0%)	-
Routine checkup	140 (24.3%)	-
Chronic treatment	128 (22.2%)	-
Acute sickness	309 (53.6%)	-
Diarrhea	-	89 (63.1%)
Fever	-	65 (46.1%)
Cough	-	95 (67.4%)
Fast breathing	-	14 (9.9%)
*Facility access*	*Mean (SD)*	*Mean (SD)*
Teaching hospitals within 1 km	0.0 (0.0)	0.0 (0.0)
General hospitals within 1 km	0.4 (0.5)	0.4 (0.5)
Private facilities within 1 km	1.8 (1.1)	1.9 (1.1)
Other health facilities within 1 km	0.9 (0.9)	1.0 (0.8)
Teaching hospitals within 5 km	0.2 (0.4)	0.2 (0.4)
General hospitals within 5 km	2.2 (0.9)	2.2 (0.8)
Private facilities within 5 km	16.0 (5.3)	16.1 (4.8)
Other health facilities within 5 km	10.8 (3.3)	10.8 (2.8)

Column (2) describes the characteristics of the adult analytic sample, which is restricted to include only adults whose most recent visit to a health facility was for care for a non-emergency condition. Column (2) describes the characteristics of the child analytic sample, which the characteristics of all children in the sampled households who sought care for diarrhea, fever, cough, or fast breathing within the past two weeks

The survey participants had a total of 402 children under-5 living in their households, of whom 141 had sought care for an episode of diarrhea (63%), fever (46%), cough (67%), or fast breathing (10%) in the past two weeks. About half (49%) of these 141 children were female.

**Fig. 1:**
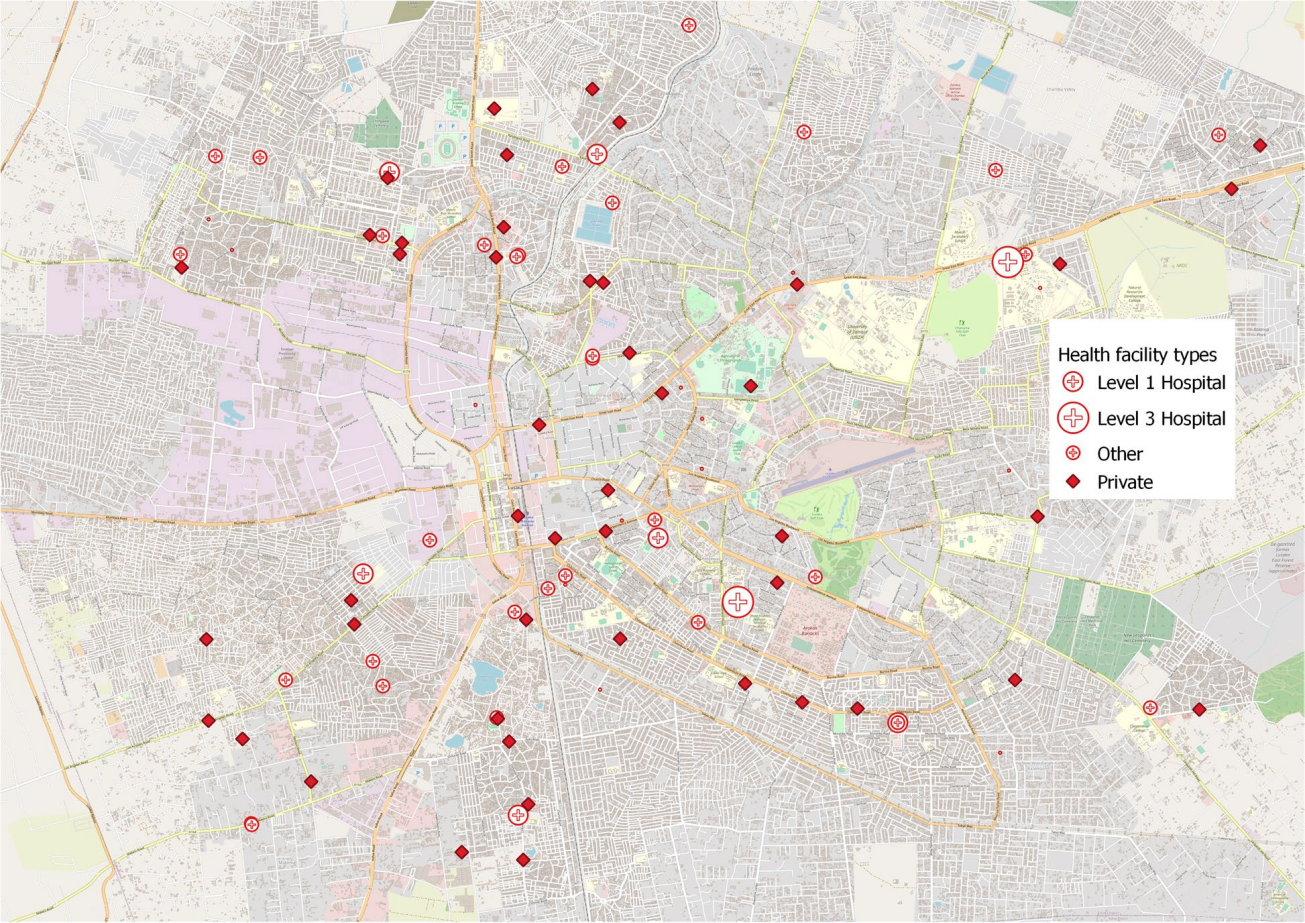
Spatial Distribution of Facilities. *Notes:* Map shows spatial distribution of health facilities within Lusaka district. “Other” facilities include health centres, health posts as well as health centers operated by missions or faith-based organizations

Figure 1 shows the spatial location of all health facilities officially recognized by the Ministry of Health within the District of Lusaka. There were a total of 88 facilities operating in Lusaka district based on the list from the Ministry of Health: two teaching hospitals, six general (Level 1) hospitals, two Level 2 hospitals, 47 private facilities and 31 smaller facilities, including health centres, health posts or mission facilities.

**Fig. 2:**
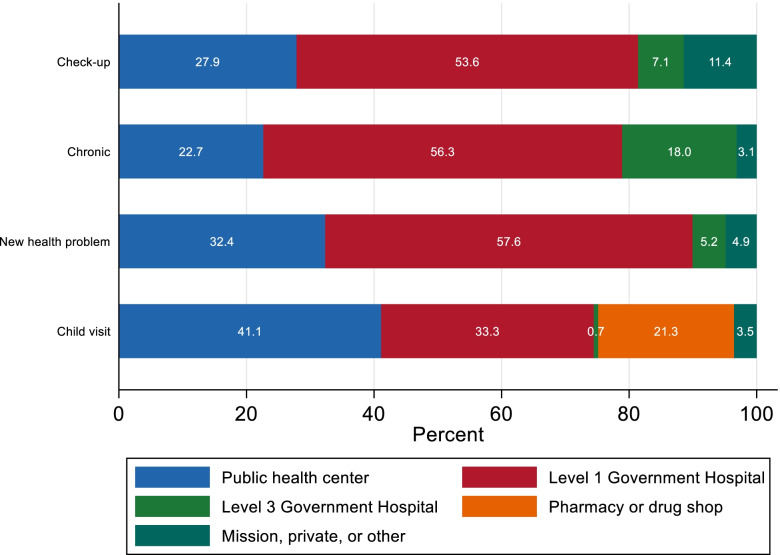
Types of facilities where people seek care, by reason for seeking care. Notes: Figure shows the percentage of respondents who sought care at different types of health facilities, by the type of health visit (adult check-up, adult chronic care visit, adult new health issue, and child visit)

Figure 2 illustrates the distribution of facilities used for care by reason for seeking care. Across all care or health problem categories, Level 1 hospitals were the most commonly used facility type, with less than one third of adult patients using health posts or health centers for checkup, chronic or acute care. Among adults, non-governmental facilities (private or faith based) were most commonly used for check–ups (11%) and teaching hospitals were most commonly used for chronic care (18%). Compared with adults, children were more likely to receive care in a health post or health center (with 41% seeking care at these facilities), or a pharmacy or drug shop (21%). One third of children received care in a hospital.

As shown in Table 3, bypassing was very common across all conditions: on average 71% (95% CI: 67% to 75%) of adults bypassed public health centres and posts, with particularly high rates for chronic conditions (77%; 95% CI: 70% to 85%). Horizontal bypassing was less common: 32% (95% CI: 29% to 36%) of adults visited a more distant rather than a nearby health facility, and this rate was similar across different reasons for health visits. Finally, the rate of two-level bypassing among adults was 8% (95% CI: 6% to 11%), with the highest observed rate for adults seeking care for chronic conditions (18%; 95% CI: 11% to 25%).

**Table 3:** Rate of bypassing, by reason for seeking care

	N	%	95% Confidence Interval
*Adults: all conditions (N* = *577)*			
Primary care bypassing	409	71%	(67% to 75%)
Horizontal bypassing	187	32%	(29% to 36%)
Two-level bypassing	49	8%	(6% to 11%)
*Adults: check-ups or preventive care (N* = *140)*			
Primary care bypassing	101	72%	(65% to 80%)
Horizontal bypassing	48	34%	(26% to 42%)
Two-level bypassing	10	7%	(3% to 11%)
*Adults: follow-up care for a chronic condition (N* = *128)*			
Primary care bypassing	99	77%	(70% to 85%)
Horizontal bypassing	47	37%	(28% to 45%)
Two-level bypassing	23	18%	(11% to 25%)
*Adults: new health issue (N* = *309)*			
Primary care bypassing	209	68%	(62% to 73%)
Horizontal bypassing	92	30%	(25% to 35%)
Two-level bypassing	16	5%	(3% to 8%)
*Children: any acute sickness (N* = *141)*			
Primary care bypassing	83	59%	(51% to 67%)
Horizontal bypassing	64	45%	(37% to 54%)
Two-level bypassing	1	1%	(0% to 2%)
*Children: diarrhea (N* = *89)*			
Primary care bypassing	53	60%	(49% to 70%)
Horizontal bypassing	40	45%	(34% to 55%)
Two-level bypassing	1	1%	(0% to 3%)
*Children: fever (N* = *65)*			
Primary care bypassing	35	54%	(41% to 66%)
Horizontal bypassing	23	35%	(23% to 47%)
Two-level bypassing	1	2%	(0% to 5%)
*Children: cough (N* = *95)*			
Primary care bypassing	55	58%	(48% to 68%)
Horizontal bypassing	47	49%	(39% to 60%)
Two-level bypassing	1	1%	(0% to 3%)
*Children: fast breathing (N* = *14)*			
Primary care bypassing	8	57%	(27% to 87%)
Horizontal bypassing	6	43%	(13% to 73%)
Two-level bypassing	1	7%	(0% to 23%)

The primary care bypassing rate among children was 59% (95% CI: 51% to 67%), slightly lower than the rate among adults. The bypassing rate was similar for children with different symptoms. The rate of horizontal bypassing was slightly higher among children than among adults at 45% (95% CI: 37% to 54%). Among children who bypassed the nearest health facility, 47% (95% CI: 35% to 59%) went to pharmacies and the remainder sought care at more distant public primary care facilities or hospitals. Finally, the rate of two-level bypassing among children was 1% (95% CI: 0% to 2%).

**Fig. 3:**
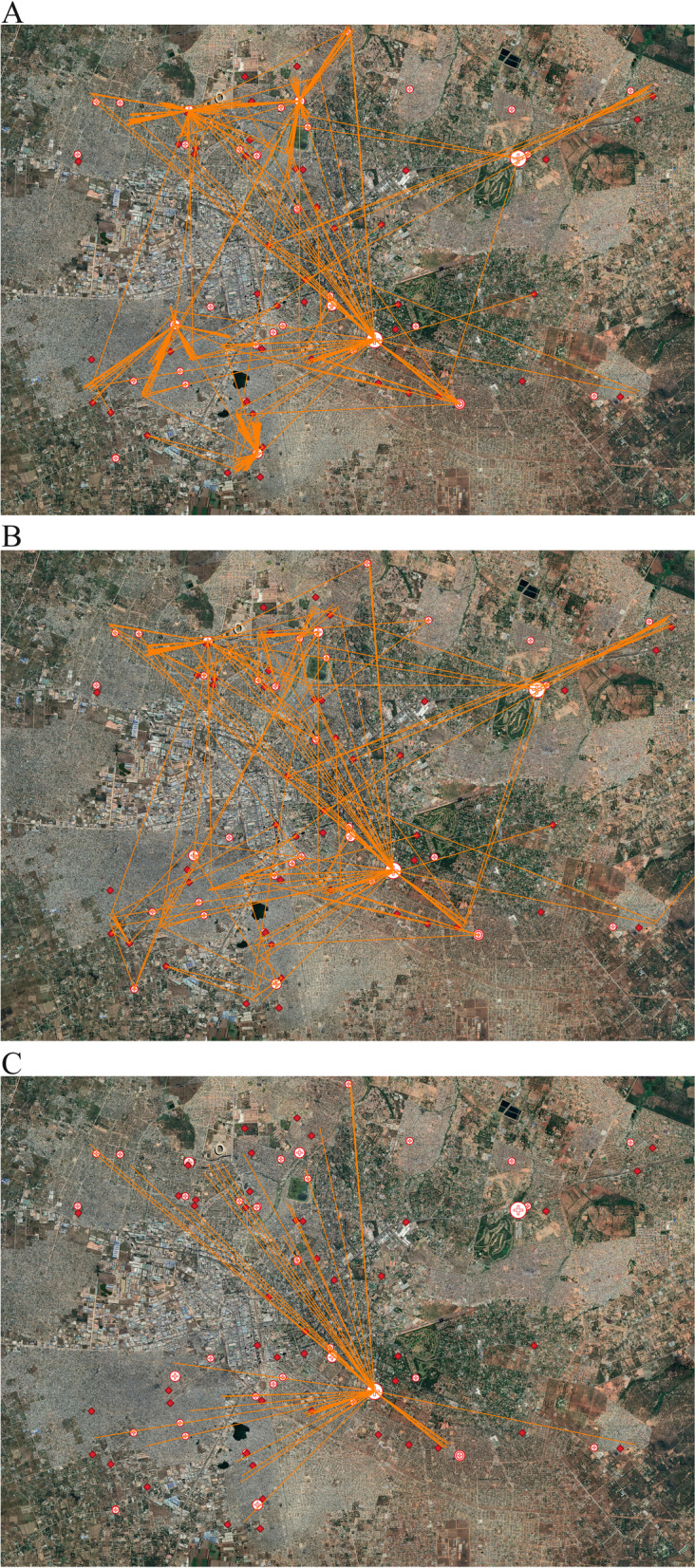
Spatial Distribution of Treatment Seeking among bypassers. Panel **A** Bypassing Health Centres and Health Posts. Panel **B** Horizontal Bypassing. Panel **C** Treatment Seeking at UTH

Figure 3 illustrates the spatial patterns of bypassing. About two thirds (67%) of the overall primary care bypassing occurs at local (Level 1) hospitals, which are located within the same constituency and thus are within two km of most households in our sample (Fig. 3, Panel A). Horizontal bypassing involves on average slightly larger distances (Fig. 3, Panel B). About half of horizontal bypassing goes to hospitals in other constituencies (UTH and Matero Level 1 hospital appears to be most popular in our sample, accounting for 20 and 14% of total horizontal bypassing, respectively) – the rest of the patients seek care at a mix of public (30%) and private or other facilities (19%) in other parts of the city. Distance travelled is on average largest for two-level bypassing, and mostly concentrated at the University Teaching Hospital (UTH) (Fig. 3, Panel C), which attracts patients from the entire city.

Bypassing rates varied significantly across the different constituencies in the sample (Additional file 1: Table S1). The rate of primary care bypassing ranged from 28 to 100%, the rate of horizontal bypassing ranged from 5 to 79%, and the rate of two-level bypassing ranged from 0 to 32% across constituencies. The large differences in care seeking behavior can be best illustrated by comparing two constituencies with very different behaviors: in one EA in Lusaka Central near Bauleni Health Centre, only 10% engaged in primary care bypassing, 15% in horizontal bypassing, and only 5% went to teaching hospitals (two-level bypassing). In contrast, in another EA near Chilenje Level 1 Hospital, the rates of bypassing were 95% (primary care bypassing), 47% (horizontal bypassing), and 32% (two-level bypassing).

As shown in Table 4 and Additional file 1: Table S2, bypassing rates varied with respondent characteristics. Among adults (Table 4), women had a 10% lower odds of primary care bypassing (95% CI: 0.83 to 0.98) and a 10% higher odds of horizontal bypassing (95% CI: 1.00 to 1.20) than men, after adjusting for other characteristics. Married participants had a 10% lower odds of horizontal bypassing (95% CI: 0.84 to 0.98) than unmarried participants, though rates of primary care bypassing and two-level bypassing were very similar between married and unmarried participants. Older respondents had higher rates of two-level bypassing and horizontal bypassing, though these associations were only statistically significant for two-level bypassing. Adults with a higher socioeconomic status as measured by education level and asset scores generally had higher rates of bypassing than those with lower socioeconomic status, though this association was not statistically significant for all outcomes and education levels. The finding (from unadjusted analyses) that two-level bypassing is more common among adults seeking care for chronic conditions than other types of care persisted after adjustment for socioeconomic characteristics.

**Table 4:** Associations between respondent characteristics and bypassing

	(1)	(2)	(3)
	Primary Care Bypassing	Two-level Bypassing	Horizontal Bypassing
Female	0.901**	0.989	1.097**
	(0.830 to 0.979)	(0.938 to 1.043)	(1.003 to 1.200)
Age (Ref = 18–29)			
30–44	1.049	1.049*	1.058
	(0.956 to 1.150)	(0.991 to 1.110)	(0.978 to 1.144)
45 +	0.987	1.052*	1.066
	(0.869 to 1.122)	(0.993 to 1.114)	(0.957 to 1.188)
Married	1.007	0.987	0.903**
	(0.934 to 1.086)	(0.927 to 1.051)	(0.835 to 0.976)
Education level (Ref = Primary or less)			
Secondary	1.072	0.999	1.106**
	(0.964 to 1.192)	(0.951 to 1.049)	(1.020 to 1.199)
Higher	1.026	1.130**	1.344***
	(0.850 to 1.238)	(1.014 to 1.259)	(1.142 to 1.581)
Asset score	1.020	1.016**	0.976
	(0.982 to 1.060)	(1.001 to 1.031)	(0.941 to 1.011)
Reason for seeking care (Ref = check-up)			
Chronic condition	1.066	1.096**	1.008
	(0.967 to 1.176)	(1.005 to 1.197)	(0.874 to 1.164)
Acute condition	0.966	0.988	0.956
	(0.885 to 1.056)	(0.946 to 1.032)	(0.860 to 1.062)
Constant	1.968***	0.991	1.335***
	(1.629 to 2.378)	(0.888 to 1.107)	(1.104 to 1.614)
Observations	577	577	577
R-squared	0.035	0.081	0.054

Table shows exponentiated coefficients and 95% confidence intervals from logistic regression models. Standard errors are clustered at the enumeration area level. “Ref” indicates the omitted reference group for categorical variables

^***^p < 0.01, **p < 0.05, *p < 0.1

Among children (Additional file 1: Table S2), primary care bypassing was higher among those whose caregivers had completed secondary education than those with primary education or less (odds ratio 1.27, 95% CI: 1.06 to 1.53), but there were no statistically significant differences by education level for two-level bypassing or horizontal bypassing. Bypassing rates also did not differ significantly by the asset quintile of the caregiver, after adjusting for other characteristics. Primary care bypassing was significantly less common for female children (odds ratio 0.78, 95% CI: 0.66 to 0.92) than male children, but other forms of bypassing did not vary significantly by gender. Bypassing rates were generally lower among children presenting with fever and higher among children presenting with diarrhea or fast breathing, though these associations were generally not statistically significant.

## Discussion

In this study, we described care-seeking patterns in Lusaka, Zambia and measured the rates of primary care bypassing, horizontal bypassing, and two-level bypassing. Despite recent government efforts to encourage use of primary care through the removal of user fees, primary care bypassing is extremely common in Lusaka, and Level 1 and Level 3 hospitals are used extensively for non-emergency care. These findings are consistent with a growing literature showing high rates of bypassing in low- and middle-income countries [20–34, 36, 37, 45–48]. Our study builds on the existing literature by mapping bypassing patterns in an urban setting. In the context of rapid urbanization in sub-Saharan Africa, where the proportion of the population living in an urban area increased from 27 to 41% over the past 30 years [1], it is important to understand care-seeking patterns in cities. Furthermore, while past studies tended to focus on a single definition of bypassing, we examined the rates of different forms of bypassing and are thus able to further understand different care-seeking patterns. While we found very high rates of primary care bypassing (71% of adults and 59% of children), we found lower rates of horizontal bypassing (26% of adults and 45% of children).

High rates of hospital use for non-emergency care, as observed in this study and others [35, 49], present a challenge for achieving the Sustainable Development Goal for universal health coverage [50]. The World Health Organization (WHO) has called for a shift of the entry point to the health system from hospitals to primary care centers to promote efficient use of resources, equitable access to care, and continuity of care [51]. In Zambia, the user fee structure is set up to discourage the use of hospitals as a first point-of-contact. While hospitals could attempt to stop this practice, it is possible that the bypassing fee incentivizes them to accept patients seeking non-emergency care.

The extensive use of pharmacies and drug shops for pediatric health care observed in this study also presents a potential challenge. Pharmacies play a significant role in primary care provision in many LMICs, often because they are considered to be convenient locations to seek care [52, 53]. However, there is evidence of important gaps in pharmacists’ education and training in many settings [52, 54], and pharmacies often lack basic medications and equipment for primary care provision [54]. Furthermore, a study in Zambia found widespread non-prescription sale of antibiotics in community pharmacies, a practice that may contribute to antimicrobial resistance [55]. It is important to understand why caregivers choose to bring their children to pharmacies instead of free public facilities. If pharmacies are to continue playing an important role in pediatric care in Zambia, there is a need to ensure that they are adequately staffed and supplied, and that measures are in place to ensure appropriate use of medication in these locations.

While this is an observational study and does not provide direct insights into reasons for bypassing, our analysis and the existing literature point to several possible explanations. First, patients may bypass because they perceive care to be of higher quality at a more distant or higher-level facility [22, 37]. In our data, these perceptions seem to vary substantially across communities: in some EAs, nearly all patients bypassed the local primary care facility while, in others, it was much more commonly used. Higher-income patients, in particular, may be willing to pay more to receive care that they perceive to be of a higher quality [29, 32, 35]; this may help explain our finding that bypassing is more common among study participants with higher levels of education and household assets. A second possible explanation is that the hours of operation of the bypassed facilities are too limited or inconvenient [56, 57], leading patients to seek care in facilities with hours that are more amenable to their schedules. Another possible explanation is that patients bypass nearby facilities due to fear of stigma from seeking care in their own communities for conditions such as HIV/AIDS. In our analysis of horizontal bypassing, we found that some patients bypassed nearby primary health centers to seek care at more distant primary health centers, while other patients bypassed nearby hospitals to seek care at more distant hospitals. The estimated HIV rate in Lusaka is 16% [58], and care-seeking for HIV/AIDS is associated with high levels of stigma [59]. Past studies in LMIC settings have found that patients may be willing to travel longer distances to avoid being recognized when seeking testing or treatment for HIV/AIDS [60, 61], so it is possible that participants in our study chose to bypass nearby facilities for this reason. Finally, many hospitals in Lusaka were upgraded from health centers in recent years [62]; it is possible that residents were unaware that they were using hospitals, though the fee structure would likely make it clear. This is an important area for future research.

The strengths of this study include the use of a dataset with a complete mapping of facilities in a major urban center that is likely representative of many urban areas in sub-Saharan Africa, and the detailed data on care-seeking behavior collected from a randomly selected household sample. These descriptive data can be used by local managers to inform analyses of bypassing behaviors and subsequently consider how to address them.

This study also has several weaknesses. First, we do not have information on whether bypassing patients were referred to higher level facilities by providers in primary health facilities, or were attending follow-up visits which can occur in specialized clinics in teaching hospitals. These care-seeking patterns would be in line with Ministry of Health guidance. While referrals and follow-up visits might help to explain the high rates of two-level bypassing by patients with chronic conditions (as 18% of patients with such conditions seek care at UTH), they are unlikely to explain the broader trends we observe in this study since we found that patients seeking care for new health conditions bypassed at only slightly lower rates than those seeking care for chronic conditions. Data on referral patterns – including whether patients were referred from primary care to higher level facilities, sought care at primary care facilities before deciding themselves to go to higher level facilities, or went straight to higher-level facilities – would help to shed further light on the challenges at the level of primary care facilities. Second, our household survey included informal sector households only. However, this is the large majority of residents in Zambia [41], and only 6.4% of the adults we approached for the study were employed in the formal sector and excluded for this reason. It seems unlikely that bypassing behavior would be less pronounced in the formal sector given the generally higher socioeconomic status of these households – assessing these differences would certainly be interesting for future studies. Third, the structure of hospital services in Zambia will be updated in 2022 as part of the 2022–2026 National Health Strategic Plan. However, the hospital mapping we used in this analysis was current for the study period and the Ministry of Health’s guidance regarding the use of primary care is not expected to change. Fourth, the time horizons are different for the child sample (past two weeks only) and the adult sample (most recent visit); this may impact our comparisons between adults and children. Finally, we make the assumption that individuals were living in their current household and were at home when they most recently sought care. If many individuals moved between when they sought care and when they were interviewed, or if they sought care during their working day, this might change our results for horizontal bypassing; however, it would not change our results for primary care bypassing or two-level bypassing.

## Conclusions

The results presented in this paper suggest that bypassing is incredibly common in Lusaka, and that existing care-seeking recommendations by the government are largely ignored. As policymakers aim to encourage the use of primary care, it is important to consider how to make lower level facilities more attractive and beneficial to patients. Hospital fee structures such as the one introduced in Zambia, whereby patients can access free primary care but have to pay to directly access care at a hospital, do not seem to deter patients from seeking care in hospitals; this suggests that patients highly value the care provided in hospitals.

## Supplementary Information

**Additional file 1:** **Figure S1.** Sample flow diagram. **Table S1.** Bypassing by study cluster. **Table S2.** Associations between respondent characteristics and bypassing among children.

**Additional file 2:** Survey instrument.

## Data Availability

The datasets used and/or analyzed during the current study will be made available on the Harvard University Dataverse upon publication.
