# Supplementary material for: Health care seeking in modern urban LMIC settings: evidence from Lusaka, Zambia
Source: BMC Public Health. 2022 Jun 16;22:1205. doi: 10.1186/s12889-022-13549-3 (PMC9202228; doi:10.1186/s12889-022-13549-3)
Supplement: Supplementary file 1 — Additional file 1: Figure S1. Sample flow diagram. Table S1. Bypassing by study cluster. Table S2. Associations between respondent characteristics and bypassing among children. [file 12889_2022_13549_MOESM1_ESM.docx]

**Supplementary Appendix to**

**“Health Care Seeking in Modern Urban LMIC Settings: Evidence from Lusaka, Zambia”**

**Figure S1: Sample flow diagram**

**
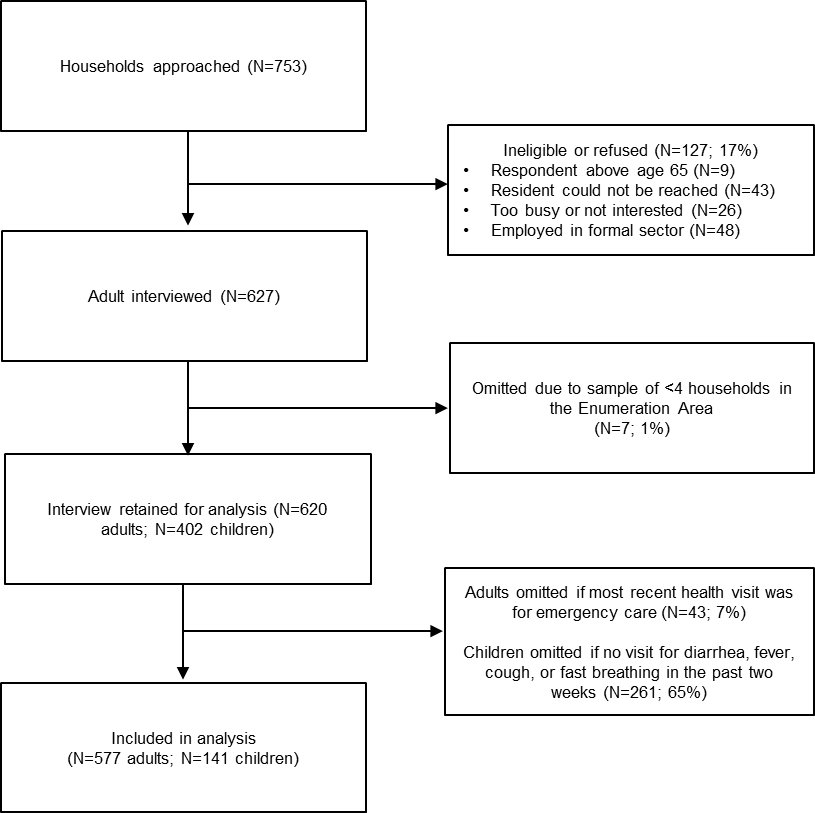
**

**Table S1: Bypassing by study cluster**

| Cluster | Constituency | Main Public Health Facility  (based on participant response) | Closest Public Health Facilities (spatially) | % Bypassing (primary) | % Bypassing (horizontal) | % Bypassing (2-levels) |
| --- | --- | --- | --- | --- | --- | --- |
| 1 | Chawama | Chawama Level 1 Hospital | Chawama Level 1 Hospital  Kuku Health Post | 90% | 30% | 10% |
| 2 | Chawama | Chawama Level 1 Hospital | Chawama Level 1 Hospital | 100% | 32% | 0% |
| 3 | Chawama | Chawama Level 1 Hospital | Chawama Level 1 Hospital | 100% | 10% | 5% |
| 4 | Chawama | Chawama Level 1 Hospital | Chawama Level 1 Hospital  Kuku Health Post | 100% | 5% | 0% |
| 5 | Chawama | Chawama Level 1 Hospital  Kuku Health Post | Kuku Health Post | 68% | 21% | 5% |
| 6 | Kabwata | Kamwala Health Centre  Kabwata Health Centre | Kamwala Health Centre  Kamwala South Health Post | 50% | 61% | 22% |
| 7 | Kabwata | UTH  Kabwata Health Centre | Kabwata Health Centre  Kamwala South Health Post | 56% | 25% | 31% |
| 8 | Kabwata | Chilenje Level 1 Hospital | Chilenje Level 1 Hospital | 95% | 47% | 32% |
| 9 | Kanyama | Kanyama Level 1 Hospital | Kanyama Level 1 Hospital | 94% | 31% | 6% |
| 10 | Kanyama | Kanyama Level 1 Hospital  Makeni Ecumenical Centre | Kanyama Level 1 Hospital | 71% | 47% | 6% |
| 11 | Kanyama | Kanyama Level 1 Hospital  Misisi Mini Hospital | Kamwala Health Centre  Kanyama Level 1 Hospital | 82% | 47% | 12% |
| 12 | Kanyama | Kanyama Level 1 Hospital | Makeni Villa Health Post | 60% | 55% | 5% |
| 13 | Kanyama | Kanyama Level 1 Hospital  The Salvation Army Men’s Clinic | Kanyama Level 1 Hospital | 82% | 24% | 0% |
| 14 | Kanyama | Kanyama Level 1 Hospital | Kanyama Level 1 Hospital | 95% | 25% | 5% |
| 15 | Lusaka Central | Bauleni Health Centre | Bauleni Health Centre | 10% | 15% | 5% |
| 18 | Matero | Matero Level 1 Hospital | Matero Level 1 Hospital | 100% | 15% | 0% |
| 19 | Matero | George Health Centre  Kapwewe Health Centre | Lilanda Health Post | 74% | 79% | 5% |
| 20 | Matero | Matero Level 1 Hospital  Matero Health Centre | Matero Health Centre | 60% | 20% | 0% |
| 21 | Matero | Matero Level 1 Hospital  Matero Health Centre | Matero Level 1 Hospital | 89% | 11% | 5% |
| 22 | Matero | Matero Health Centre | Matero Health Centre | 26% | 47% | 11% |
| 23 | Matero | Matero Level 1 Hospital | Matero Level 1 Hospital | 89% | 44% | 22% |
| 24 | Matero | George Health Centre  Paradise Health Post | Paradise Health Post | 28% | 39% | 11% |
| 25 | Munali | Chelstone Health Centre | Chelstone Health Centre | 50% | 56% | 25% |
| 27 | Mandevu | Chipata Level 1 Hospital | Chipata Level 1 Hospital  Mandevu Health Centre | 95% | 10% | 10% |
| 28 | Mandevu | Chipata Level 1 Hospital  Kabanana Health Post | Mandevu Health Centre | 78% | 22% | 11% |
| 29 | Mandevu | Chipata Level 1 Hospital  Chaisa Health Centre | Kabanana Health Post | 70% | 35% | 10% |
| 30 | Mandevu | Chipata Level 1 Hospital  Chaisa Health Centre | Chaisa Health Centre  Garden Shimizu Health Post | 42% | 26% | 5% |
| 31 | Mandevu | Chipata Level 1 Hospital | Chaisa Health Centre | 89% | 28% | 6% |
| 32 | Mandevu | Chipata Level 1 Hospital | Garden Shimizu Health Post | 72% | 28% | 0% |
| 33 | Mandevu | Matero Level 1 Hospital  Mandevu Health Centre | Chipata Level 1 Hospital | 61% | 50% | 6% |
| 34 | Mandevu | Chifundo Clinic  Chaisa Health Centre | Chipata Level 1 Hospital | 20% | 30% | 0% |

Notes: Several constituencies have two facilities listed as “closest public facilities (spatially).” This is because different facilities were spatially closest to different respondents within the cluster.

Table S2: Associations between respondent characteristics and bypassing among children

|  | (1) | | (2) | (3) | |
| --- | --- | --- | --- | --- | --- |
|  | Primary care bypassing | Two-level bypassing | | | Horizontal bypassing |
|  |  |  | | |  |
| Caregiver education (Ref = Primary or less) |  |  | | |  |
| Secondary | 1.273** | 0.995 | | | 1.127 |
|  | (1.063 - 1.525) | (0.985 - 1.005) | | | (0.921 - 1.377) |
| Higher | 1.016 | 1.107 | | | 1.108 |
|  | (0.596 - 1.730) | (0.938 - 1.306) | | | (0.811 - 1.513) |
| Asset quintile | 1.029 | 1.007 | | | 0.956 |
|  | (0.954 - 1.111) | (0.997 - 1.017) | | | (0.873 - 1.046) |
| Reason for seeking care |  |  | | |  |
| Diarrhea | 1.072 | 1.015 | | | 1.077 |
|  | (0.852 - 1.350) | (0.988 - 1.043) | | | (0.863 - 1.344) |
| Fever | 0.905 | 0.993 | | | 0.841* |
|  | (0.728 - 1.125) | (0.982 - 1.005) | | | (0.689 - 1.025) |
| Cough | 0.977 | 1.010 | | | 1.155 |
|  | (0.822 - 1.162) | (0.979 - 1.041) | | | (0.951 - 1.403) |
| Fast breathing | 1.081 | 1.062 | | | 1.017 |
|  | (0.809 - 1.443) | (0.974 - 1.158) | | | (0.790 - 1.310) |
| Female child | 0.775*** | 1.011 | | | 0.957 |
|  | (0.657 - 0.915) | (0.991 - 1.031) | | | (0.752 - 1.217) |
| Constant | 1.713*** | 0.962 | | | 1.606*** |
|  | (1.267 - 2.317) | (0.898 - 1.031) | | | (1.157 - 2.228) |
|  |  |  | | |  |
| Observations | 141 | 141 | | | 141 |
| R-squared | 0.162 | 0.179 | | | 0.067 |
| Notes: Table shows exponentiated coefficients and 95% confidence intervals from logistic regression models. Standard errors are clustered at the enumeration area level. “Ref” indicates the omitted reference group for categorical variables. *** p<0.01, ** p<0.05, * p<0.1 | | | | | |
