# Supplementary material for: Health care seeking in modern urban LMIC settings: evidence from Lusaka, Zambia
Source: BMC Public Health. 2022 Jun 16;22:1205. doi: 10.1186/s12889-022-13549-3 (PMC9202228; doi:10.1186/s12889-022-13549-3)
Supplement: Supplementary file 2 — Additional file 2: Survey instrument. [file 12889_2022_13549_MOESM2_ESM.pdf]

Survey

Survey

Survey details

[interviewer_id]
Interviewer name

Choose only one option:

○	Gracious
○	Sitwala
○	Ruth
○	Mundai

[start_time]

Start time:

[end_time]

End time:

[store_gps]
Collect the GPS coordinates of this store.

Geopoint:

[selected_ward]
Name of ward

Choose only one option:

○	Chaisa
○	Chakunkula
○	Chawama
○	Chilenje
○	Harry Mwanga Nkumbula
○	Idependence
○	Justin Kabwe
○	Kabulonga
○	Kabwata
○	Kamwala
○	Kanyama
○	Kapwepwe
○	Lilayi
○	Lima
○	Lubwa
○	Matero
○	Muchinga
○	Munali
○	Mwembeshi
○	Ngwerere
○	Nkoloma
○	Raphael
○	Roma

[selected_ea]
Enumeration area

The choice list of this question (comprising 35 choices) was replaced with a text field due to this question having choice filter and more than 10 choices.

[audit]

Audit:

Socio-demographics

[Q0hh]
Q0. Head of household?

Choose only one option:

○	Yes
○	No, spouse of head

[Q1age]
Q1. Respondent's age?

[Q2sex]
Q2. Record sex as observed

Choose only one option:

○	Female
○	Male

[Q3marital_stat]
Q3. What is your current marital status?

Choose only one option:

○	Married
○	Single
○	Divorced
○	Cohabiting
○	Separated
○	Widow

[Q4edn_lev]
Q4. What is your highest level of education completed?

Choose only one option:

○	No, formal schooling
○	Some primary completed
○	Primary school completed
○	Some secondary completed
○	Secondary school completed
○	Some college or university completed
○	College or University completed
○	Postgraduated completed

[Q5rlgn]
Q5. What is your religion?

Choose only one option:

○	Catholic
○	Protestant
○	Muslim
○	Other

• Relevant when:
Q5. What is your religion? = Other.

[Q5rlgn_oth]
Q5a. Specify other

Household characteristics

[Q6drkn_sour]
Q6. What is the main source of drinking water for members of this household?

Choose only one option:

○	Piped into dwelling
○	Piped into yard/plot
○	Piped to neighbor
○	Public tab/standpipe
○	Borehole
○	Protected well
○	Unprotected well
○	Protected spring
○	Unprotected spring
○	Rainwater
○	Tanker truck
○	Surface water (river/dam/lake/pond)
○	Bottled water
○	Other

• Relevant when:
Q6. What is the main source of drinking water for members of this household? = Other.

[Q6drkn_sour_oth]
Q6a. Specify other

• Relevant when:
Q6. What is the main source of drinking water for members of this household? = Bottled water.

[Q7other_wter_sour]
Q7. What is the main source of water used by your household for other purposes such as cooking and handwashing?

Choose only one option:

○	Piped into dwelling
○	Piped into yard/plot
○	Piped to neighbor
○	Public tab/standpipe
○	Borehole
○	Protected well
○	Unprotected well
○	Protected spring
○	Unprotected spring
○	Rainwater
○	Tanker truck
○	Surface water (river/dam/lake/pond)
○	Bottled water
○	Other

• Relevant when:
Q7. What is the main source of water used by your household for other purposes such as cooking and handwashing? = Other.

[Q7other_wter_sour_oth]
Q7a. Specify other

• Relevant when:
Q6. What is the main source of drinking water for members of this household? ≠ Piped into dwelling || Q6. What is the main source of drinking water for members of this household? = Piped into yard/plot || Q6. What is the main source of drinking water for members of this household? = Piped to neighbor || Q6. What is the main source of drinking water for members of this household? = Bottled water.

[Q8water_lcn]
Q8. Where is the water source located?

Choose only one option:

○	In your own dwelling
○	In your own yard/plot
○	Elsewhere

• Relevant when:
Q8. Where is the water source located? = Elsewhere.

[Q9water_time]
Q9. How long does it take to get there, wait, get water and come back? Minutes

• Relevant when:
Q6. What is the main source of drinking water for members of this household? = Piped into dwelling || Q6. What is the main source of drinking water for members of this household? = Piped into yard/plot || Q6. What is the main source of drinking water for members of this household? = Piped to neighbor || Q6. What is the main source of drinking water for members of this household? = Public tab/standpipe || Q6. What is the main source of drinking water for members of this household? = Borehole || Q7. What is the main source of water used by your household for other purposes such as cooking and handwashing? = Piped into dwelling || Q7. What is the main source of water used by your household for other purposes such as cooking and handwashing? = Piped into yard/plot || Q7. What is the main source of water used by your household for other purposes such as cooking and handwashing? = Piped to neighbor || Q7. What is the main source of water used by your household for other purposes such as cooking and handwashing? = Public tab/standpipe || Q7. What is the main source of water used by your household for other purposes such as cooking and handwashing? = Borehole.

[Q11water_avbty]
Q11. In the past two weeks, was the water from this source not available for at least one full day?

Choose only one option:

○	Yes
○	No
○	Don't know

• Relevant when:
Q6. What is the main source of drinking water for members of this household? ≠ Piped into dwelling || Q6. What is the main source of drinking water for members of this household? = Piped into yard/plot || Q6. What is the main source of drinking water for members of this household? = Piped to neighbor || Q6. What is the main source of drinking water for members of this household? = Public tab/standpipe || Q6. What is the main source of drinking water for members of this household? = Bottled water || Q6. What is the main source of drinking water for members of this household? = Borehole || Q7. What is the main source of water used by your household for other purposes such as cooking and handwashing? = Piped into dwelling || Q7. What is the main source of water used by your household for other purposes such as cooking and handwashing? = Piped into yard/plot || Q7. What is the main source of water used by your household for other purposes such as cooking and handwashing? = Piped to neighbor || Q7. What is the main source of water used by your household for other purposes such as cooking and handwashing? = Public tab/standpipe || Q7. What is the main source of water used by your household for other purposes such as cooking and handwashing? = Borehole.

[Q12safe_wter]
Q12. Do you do anything to the water to make it safer to drink?

Choose only one option:

○	Yes
○	No
○	Don't know

• Relevant when:
Q12. Do you do anything to the water to make it safer to drink? = Yes.

[Q13mk_safe]
Q13. What do you usually do to make the water safer to drink?

Choose one or more options:

☐	Boil
☐	Add bleach/chlorine
☐	Strain through cloth
☐	Use water filter(ceramic, sand, composite, etc)
☐	Solar disinfection
☐	Let it stand and settle
☐	Other
☐	Don't know

• Relevant when:
Q13. What do you usually do to make the water safer to drink? = Don't know.

[Q13mk_safe_oth]
Q13a. Specify other

[Q14str_water]
Q14. How do you store your drinking water?

Choose only one option:

○	Closed container/jerry can
○	Open container/bucket
○	Does not store water
○	Other

• Relevant when:
Q14. How do you store your drinking water? = Other.

[Q14str_water_oth]
Q14a. Specify other

Toilet facility

[Q15toilet_typ]
Q15. What kind of toilet facility do members of this household usually use?

Choose only one option:

○	Flush to piped sewer system
○	Flush to septic tank
○	Flush to pit latrine
○	Flush to somewhere else
○	Flush, Don’t know where
○	Ventilated improved pit latrine
○	Pit latrine with slab
○	Pit latrine without slab/Open pit
○	Composting toilet
○	Bucket toilet
○	Hanging toilet/hanging latrine
○	No facility/bush/field
○	Other

• Relevant when:
Q15. What kind of toilet facility do members of this household usually use? = Other.

[Q15toilet_typ_oth]
Q15a. Specify other

• Relevant when:
Q15. What kind of toilet facility do members of this household usually use? ≠ No facility/bush/field.

[Q16share_toilet]
Q16. Do you share this facility with other households?

Choose only one option:

○	Yes
○	No

• Relevant when:
Q16. Do you share this facility with other households? = Yes.

[Q17hh_toilet]
Q17. Including your own household, how many households use this toilet facility?

• Relevant when:
Q15. What kind of toilet facility do members of this household usually use? ≠ No facility/bush/field.

[Q18toilet_lcn]
Q18. Where is this toilet facility located?

Choose only one option:

○	In your own dwelling
○	In your own yard/plot
○	Elsewhere

Cooking details

[Q19fuel_ckn]
Q19. What type of fuel does your household mainly use for cooking?

Choose only one option:

○	Electricity
○	Solar Power
○	Liquid Propane (LP)
○	Natural Gas
○	Biogas
○	Kerosene
○	Coal, Lignite
○	Charcoal
○	Wood
○	Straw/Shrubs/Grass
○	Agricultural Crop
○	Animal Dung
○	No food cooked in household
○	Other

• Relevant when:
Q19. What type of fuel does your household mainly use for cooking? = Wood.

[Q19fuel_ckn_oth]
Q19a. Specify other

• Relevant when:
Q19. What type of fuel does your household mainly use for cooking? ≠ No food cooked in household.

[Q20ckn_lcn]
Q20. Is the cooking usually done in the house, in a separate building or outdoors?

Choose only one option:

○	In the house
○	In a separate building
○	Outdoors
○	Other

• Relevant when:
Q20. Is the cooking usually done in the house, in a separate building or outdoors? = Answer "96" not found;.

[Q20ckn_lcn_oth]
Q20a. Specify other

• Relevant when:
Q20. Is the cooking usually done in the house, in a separate building or outdoors? = In the house.

[Q21sp_ktn]
Q21. Do you have a separate room, which is used, as a kitchen?

Choose only one option:

○	Yes
○	No

Household possession

[Q22slp_room]
Q22. How many rooms in this household are used for sleeping?

[Q23a_electric]
Q23a.Does your household have electricity?

Choose only one option:

○	Yes
○	No

[Q2b_internet]
Q23b.Does your household have access to internet?

Choose only one option:

○	Yes
○	No

[Q23c_bed]
Q23c.Does your household have a bed?

Choose only one option:

○	Yes
○	No

[Q23d_table]
Q23d.Does your household have a table?

Choose only one option:

○	Yes
○	No

[Q23e_sofa]
Q23e.Does your household have a sofa?

Choose only one option:

○	Yes
○	No

[Q23f_radio]
Q23f.Does your household have a radio?

Choose only one option:

○	Yes
○	No

[Q23g_tele]
Q23g.Does your household have a television?

Choose only one option:

○	Yes
○	No

[Q23h_compt]
Q23h.Does your household have a computer/laptop?

Choose only one option:

○	Yes
○	No

[Q23i_fridge]
Q23i.Does your household have a refrigerator?

Choose only one option:

○	Yes
○	No

[Q23j_washmach]
Q23j.Does your household have a washing machine?

Choose only one option:

○	Yes
○	No

[Q23k_aircon]
Q23k.Does your household have an air conditioner?

Choose only one option:

○	Yes
○	No

[Q23l_genet]
Q23l.Does your household have a generator?

Choose only one option:

○	Yes
○	No

[Q23m_wave]
Q23m.Does your household have a microwave?

Choose only one option:

○	Yes
○	No

[Q23n_heater]
Q23n.Does your household have a geyser?

Choose only one option:

○	Yes
○	No

[Q24a_watch]
Q24a.Does any member of this household own a watch?

Choose only one option:

○	Yes
○	No

[Q24b_phone]
Q24b.Does any member of this household own a mobile phone?

Choose only one option:

○	Yes
○	No

[Q24c_bic]
Q24c.Does any member of this household own a bicycle?

Choose only one option:

○	Yes
○	No

[Q24d_motor]
Q24d.Does any member of this household own a motorcyle or scooter?

Choose only one option:

○	Yes
○	No

[Q24e_car]
Q24e.Does any member of this household own a car or truck?

Choose only one option:

○	Yes
○	No

[Q25bank]
Q25.Does any member of this household own a bank account?

Choose only one option:

○	Yes
○	No

Materials of housing

[Q26flr_mat]
Q26.Observe the main material of the floor of the dwelling

Choose only one option:

○	Earth/sand
○	Dung
○	Wood Planks
○	Palm/Bamboo
○	Wooden floor
○	Vinyl (PVC)
○	Ceramic/terazzo tiles
○	Cement
○	Carpet
○	Other

• Relevant when:
Q26.Observe the main material of the floor of the dwelling = Other.

[Q26flr_mat_other]
Q26a.Specify other

[Q27rof_mat]

Q27.Observe the main material of the roof of the dwelling

Choose only one option:

○	No roof
○	Thatch/Palm leaf
○	Rustic Mat
○	Palm/Bamboo
○	Wood Planks
○	Cardboard
○	Metal/Iron sheets
○	Wood
○	Calamine/Cement Fiber
○	Ceramic Tiles/Harvey tiles
○	Cement
○	Roofing shingles
○	Mud tiles
○	Asbestos
○	Other

• Relevant when:
Q27.Observe the main material of the roof of the dwelling = Other.

[Q27rof_mat_other]

Q27a.Specify other

[Q28type_wall]
Q28.Observe the main material of the exterior walls of the dwelling

Choose only one option:

○	No walls
○	Cane/Palm/Trunks
○	Mud tiles
○	Mudbrick
○	Bamboo with mud
○	Stone with mud
○	Plywood
○	Cardboard
○	Reused wood
○	Cement
○	Stone with lime/Cement
○	Burned bricks
○	Cement blocks
○	Wood planks/shingles
○	Other

• Relevant when:
Q28.Observe the main material of the exterior walls of the dwelling = Other.

[Q28type_wall_other]
Q28a.Specify other

Overall health status

[Q29hlth_status]
Q29.Overall, how would you rate your health status

Choose only one option:

○	Very good
○	Good
○	Moderate
○	Bad
○	Very bad

Health facility visit

[Q30visit_outpt]
Q30.In the past 12 months, how many times did you seek care at a clinic or hospital? (only for outpatient visit)

[Q31last_visit]
Q31.How long ago was your last visit to a clinic or hospital?

[Q32reson_visit]
Q32.What was the main reason for your last visit?

Choose only one option:

○	Emergency, Accident or Injury
○	Check up or preventive care
○	Follow up on existing chronic problem
○	New health issue

[Q33nme_fac]
Q33.What was the name of the facility?

[Q33a_nbhd_fac]
Q3a.Which neighborhood is the facility located in?

[Q33b_typ_fac]
Q33b.What type of health facility was it?

Choose only one option:

○	Government Health center
○	Government Hospital
○	Mission hospital
○	Pharmacy
○	Private clinic/hospital
○	Don’t know
○	Other

• Relevant when:
Q33b.What type of health facility was it? = Other.

[Q33b_typ_fac_other]
Q33ba.Specify other

[Q33c_txt_spend]
Q33c.Overall, how much did you spend for the treatment of the illness (including drugs at the pharmacies)?

Thinking about your last health facility visit (not including overnight stays) how would you rate the following:

[Q34length_wait]
Q34.The length of time you waited before you were seen

Choose only one option:

○	Excellent
○	Very good
○	Good
○	Fair
○	Poor

[Q35provid_listen]
Q35.Whether the health provider listened to you carefully

Choose only one option:

○	Excellent
○	Very good
○	Good
○	Fair
○	Poor

[Q36provider_knlg]
Q36.The health provider’s medical knowledge and skills

Choose only one option:

○	Excellent
○	Very good
○	Good
○	Fair
○	Poor

[Q37provider_respt]
Q37.The level of respect the provider showed you

Choose only one option:

○	Excellent
○	Very good
○	Good
○	Fair
○	Poor

[Q38provider_time]
Q38.The amount of time the provider spent with you in the visit.

Choose only one option:

○	Excellent
○	Very good
○	Good
○	Fair
○	Poor

[Q39overall_care]
Q39.Overall, considering everything, how would you rate the quality of care you received?

Choose only one option:

○	Excellent
○	Very good
○	Good
○	Fair
○	Poor

[Q40overall_visit]
Q40.Overall, thinking about your entire visit, how would you rate the care you received?

Choose only one option:

○	Excellent
○	Very good
○	Good
○	Fair
○	Poor

Child health

[Q41has_child]
Q41.Are there any children under age five living in this household?

Choose only one option:

○	Yes
○	No

• Relevant when:
Q41.Are there any children under age five living in this household? = Yes.

[Q41a_childn]
Q41a.How many are there?

• Relevant when:
Q41.Are there any children under age five living in this household? = Yes.

child info

Question	Element 1	Element 2	Element 3	Element 4	Element 5
[Q42name_child]<br>**Q42.Name of child**					
[Q42sex_child]<br>**Q42.Sex of child**<br>Choose only one option:<br>Female<br>Male					
[Q42date_child]<br>**Q42.Date of birth**<br>Date: __ / __ / ____ (DD/MM/YYYY)					
[Q43diarrhea]<br>**Q42.Has (name) had diarrhea at any time in the last 2 weeks?**<br>Choose only one option:<br>Yes<br>No<br>Don't know					

Question	Element 1	Element 2	Element 3	Element 4	Element 5
[Q44fever]<br>**Q44.Has (name) been ill with a fever any time in the last 2 weeks?**<br>Choose only one option:<br>Yes<br>No<br>Don't know					
[Q45cough]<br>**Q45.Has (name) had an illness with a cough at any time in the last 2 weeks?**<br>Choose only one option:<br>Yes<br>No<br>Don't know					
[Q46fastbreath]<br>**Q45.Has (name) had fast, short, rapid breaths or difficulty breathing at any time in the last 2 weeks?**<br>Choose only one option:<br>Yes<br>No<br>Don't know					
• Relevant when:<br>Q42.Has (name) had diarrhea at any time in the last 2 weeks? = Yes || Q44.Has (name) been ill with a fever any time in the last 2 weeks? = Yes || Q45.Has (name) had an illness with a cough at any time in the last 2 weeks? = Yes.<br><br>[Q47advice_chd]<br>**Q47.Did you seek advice or treatment for the illness from any source?**<br>Choose only one option:<br>Yes<br>No<br>Don't know					

Question	Element 1	Element 2	Element 3	Element 4	Element 5
• Relevant when: Q47.Did you seek advice or treatment for the illness from any source? = Yes.<br><br>[Q48name_trxtchd]<br>**Q48.What was the name of the place you sought treatment?**					
• Relevant when: Q47.Did you seek advice or treatment for the illness from any source? = Yes.<br><br>[Q49typfac_chd]<br>**Q49.What type of facility was it?**<br><br>Choose only one option:<br>Government Health center<br>Government Hospital<br>Mission hospital<br>Pharmacy<br>Private clinic/hospital<br>Don't know<br>Other					
• Relevant when: Q47.Did you seek advice or treatment for the illness from any source? = Yes.<br><br>[Q50nbhd_child]<br>**Q50.What neighborhood is it located in?**					

Question	Element 1	Element 2	Element 3	Element 4	Element 5
• Relevant when: Q47.Did you seek advice or treatment for the illness from any source? = Yes.<br><br>[Q51antimal_drug]<br>**Q51.At any time during the illness, did (Name) get antimalarial for the illness?**<br>Choose only one option:<br>Yes<br>No<br>Don't know					
• Relevant when: Q47.Did you seek advice or treatment for the illness from any source? = Yes.<br><br>[Q52antibio_drug]<br>**Q52.At any time during the illness, did (Name) get antibiotics for the illness?**<br>Choose only one option:<br>Yes<br>No<br>Don't know					
• Relevant when: Q47.Did you seek advice or treatment for the illness from any source? = Yes.<br><br>[Q52atrxtcost]<br>**052.Overall, how much did you spend for the treatment of the illness (including drugs at the pharmacies)?**					

General facility access

[Q53nbd_par]

Q53.What is the name of the closest drug shop or pharmacy from your house?

• Relevant when:
Q53.What is the name of the closest drug shop or pharmacy from your house? ≠ 88.

[Q53a_nbhd_phar]
Q53a.Which neighborhood is this drug shop or pharmacy located?

• Relevant when:
Q53.What is the name of the closest drug shop or pharmacy from your house? ≠ 88.

[Q53b_walk_phar]
Q53b.How long does it take to walk from your house to the drug shop or pharmacy? (assuming you don't stop and walk fast) (minutes)

[Q54privt_fac]
Q54.What is the name of the closest private health facility from your house?

• Relevant when:
Q54.What is the name of the closest private health facility from your house? ≠ 88.

[Q54a_nbhd_priv]
Q54a.Which neighborhood is this private health facility located?

• Relevant when:
Q54.What is the name of the closest private health facility from your house? ≠ 88.

[Q54b_walk_priv]
Q54b.How long does it take walk from here to this private health facility? (assuming you don't stop and walk fast) (minutes)

[Q55_govtfac]
Q55.What is the name of the closest government health facility from your house?

• Relevant when:
Q55.What is the name of the closest government health facility from your house? ≠ 88.

[Q55nbhd_govtfac]
Q55a.Which neighborhood is this government health facility located?

• Relevant when:
Q55.What is the name of the closest government health facility from your house? ≠ 88.

[Q55b_walk_govtfac]
Q55b.How long does it take to walk from here to this government facility? (assuming you don't stop and walk fast)

[Q56month_expd]
Q56.On an average, how much does your household spend each month on healthcare (all members combined, including drugs, treatment and transport)?

[Q57lge_expd]
Q57.What was the largest health-related expenditure your household had last year and how much did you pay in total, including drugs, treatment and transport?

• Relevant when:
Q57.What was the largest health-related expenditure your household had last year and how much did you pay in total, including drugs, treatment and transport? > 0.

[Q57a_med_prob]
Q57a.What was the problem?

• Relevant when:
Q57.What was the largest health-related expenditure your household had last year and how much did you pay in total, including drugs, treatment and transport? > 0.

[Q57b_date_medprob]
Q57b.When did this occur?

Date: __ / __ / ____ (DD/MM/YYYY)

• Relevant when:
Q57.What was the largest health-related expenditure your household had last year and how much did you pay in total, including drugs, treatment and transport? > 0.

[Q57c_txrtcost]
Q57c.What was the total treatment cost? (cost of diagnosis, treatment and drugs)

[Q57d_pharm]
Q57d. How much of this money did you spend at the pharmacy or private drug store?

[Q57e_diagnos]
Q57e. How much of this money did you spend for diagnostics elsewhere?

[O57f_hf]
Q57f. How much of this money did you spend at the actual health facility?

[Q57g_nonhthcost]
Q57g. What was the total cost of non-health related cost? (Transport)

• Relevant when:
Q57.What was the largest health-related expenditure your household had last year and how much did you pay in total, including drugs, treatment and transport? > 0.

[Q57h_make_paymt]
Q57h.How did you make the payment?

Choose only one option:

○	Paid myself
○	Borrowed from family or friends
○	Sold assets
○	Did not pay

Confidence and trust

[Q58confid_pub]
Q58.How confident are you that if you become very sick tomorrow, you would be able to receive effective treatment from the public health facilities?

Choose only one option:

○	Not at all confident
○	Not very confident
○	Somewhat confident
○	Very confident

[Q59confid_priv]
Q59.How confident are you that if you become very sick tomorrow, you would be able to receive effective treatment from the private health facilities?

Choose only one option:

○	Not at all confident
○	Not very confident
○	Somewhat confident
○	Very confident

[Q60trust_govt]
Q60.How much of the time do you think you can trust the National government to do what is right?

Choose only one option:

○	Always
○	Most of the time
○	Some of the time
○	Hardly ever
○	Never

[Q61hlthsec_rate]
Q61.How well or badly is the health sector doing in improving health services?

Choose only one option:

○	Very badly
○	Fairly badly
○	Fairly well
○	Very well
○	Don’t know or I have never heard

Political affiliation

[Q62presid_elec]
Q62.If presidential elections were tomorrow, would you like a change in government?

Choose only one option:

○	Yes
○	No
○	Don't know
○	Refused

[Q63rate_govt]

Q63.How well do you think the current government doing in performing their duties?

Choose only one option:

○	Very badly
○	Fairly badly
○	Fairly well
○	Very well
○	Don't know or I have never heard

Health insurance

[Q64_insur]

Q64. Zambia has launched the National Health Insurance for Zambian Households to access health services without financial hardship. Do you currently have any health insurance

Choose only one option:

○	Yes
○	No

• Relevant when:

Q64. Zambia has launched the National Health Insurance for Zambian Households to access health services without financial hardship. Do you currently have any health insurance = Yes.

[Q64a_typ]

Q64a.What type of health insurance

Choose only one option:

○	National Health Insurance
○	Private health insurance
○	Employer-based
○	Community-based/Mutual Health Insurance
○	Other

• Relevant when:

Q64a.What type of health insurance = Other.

[Q64b_typ_oth]

Q64b.Specify other

• Relevant when:
Q64. Zambia has launched the National Health Insurance for Zambian Households to access health services without financial hardship. Do you currently have any health insurance = Yes.

[Q64c_card]
Q64c.May I please see your insurance card?

Choose only one option:

○	Yes, card observed
○	No refused
○	Did not have it

• Relevant when:
Number drawn for experiemental selection ≤ 0.5.

[notselected]
Note: Household was not selected for insurance game

• Relevant when:
Number drawn for experiemental selection > 0.5 && Q64. Zambia has launched the National Health Insurance for Zambian Households to access health services without financial hardship. Do you currently have any health insurance = No.

Health insurance Exercise

[Q65game]
Q65.We have developed a little game that illustrates how insurance works in practice. Would you be willing to play a few rounds of this game with me?”

Choose only one option:

○	Yes
○	No

• Relevant when:
Q65.We have developed a little game that illustrates how insurance works in practice. Would you be willing to play a few rounds of this game with me?” = Yes.

[r1_healthy]
Game 1: how many of the 12 cards drawn were healthy cards?

• Relevant when:
Q65.We have developed a little game that illustrates how insurance works in practice. Would you be willing to play a few rounds of this game with me?” = Yes.

[r1_kids]
..how many child sickness?

• Relevant when:
Q65.We have developed a little game that illustrates how insurance works in practice. Would you be willing to play a few rounds of this game with me?” = Yes.

[r1_physio]
..how many physio events?

• Relevant when:
Q65.We have developed a little game that illustrates how insurance works in practice. Would you be willing to play a few rounds of this game with me?” = Yes.

[r1_unknown]
..how many unknown problems?

• Relevant when:
Q65.We have developed a little game that illustrates how insurance works in practice. Would you be willing to play a few rounds of this game with me?” = Yes.

[r1_major]
..how many major events?

• Relevant when:
Q65.We have developed a little game that illustrates how insurance works in practice. Would you be willing to play a few rounds of this game with me?” = Yes.

[r2_healthy]
Game 2: how many of the 12 cards drawn were healthy cards?

• Relevant when:
Q65.We have developed a little game that illustrates how insurance works in practice. Would you be willing to play a few rounds of this game with me?” = Yes.

[r2_kids]
..how many child sickness?

• Relevant when:
Q65.We have developed a little game that illustrates how insurance works in practice. Would you be willing to play a few rounds of this game with me?” = Yes.

[r2_physio]
..how many physio events?

• Relevant when:
Q65.We have developed a little game that illustrates how insurance works in practice. Would you be willing to play a few rounds of this game with me?” = Yes.

[r2_unknown]
..how many unknown problems?

• Relevant when:
Q65.We have developed a little game that illustrates how insurance works in practice. Would you be willing to play a few rounds of this game with me?” = Yes.

[r2_major]
..how many major events?

• Relevant when:
Q64. Zambia has launched the National Health Insurance for Zambian Households to access health services without financial hardship. Do you currently have any health insurance = No.

[Q66pay]
Q66.How much would you be willing to pay to get health insurance per month for yourself?

• Relevant when:
Q64. Zambia has launched the National Health Insurance for Zambian Households to access health services without financial hardship. Do you currently have any health insurance = No.

[Q66a_pay]
Q66a.How much would you be willing to pay to get health insurance per month for entire household?

• Relevant when:
Q66a.How much would you be willing to pay to get health insurance per month for entire household? > 0.

[Q66b.hhnum]
Q66b. How many dependants do you have?

• Relevant when:
Q64. Zambia has launched the National Health Insurance for Zambian Households to access health services without financial hardship. Do you currently have any health insurance = No && Q66a.How much would you be willing to pay to get health insurance per month for entire household? > 0.

[Q67_fees]
Q67.Would you rather pay monthly fees or annual fees?

Choose only one option:

○	Monthly
○	Annually
○	Other

• Relevant when:
Q67.Would you rather pay monthly fees or annual fees? = Other.

[Q67a_oth]
Q67a.Other specify

• Relevant when:
Q67.Would you rather pay monthly fees or annual fees? = Annually.

[Q67a_ann]
Q67b. If annual, which month would you prefer to pay the annual fee?

Choose only one option:

○	January
○	Februrary
○	March
○	April
○	May
○	June
○	July
○	August
○	September
○	October
○	November
○	December

• Relevant when:
Q66.How much would you be willing to pay to get health insurance per month for yourself? > 0.

[Q68]
Q68.Based on the 1% of your monthly income, how much will you pay for health insurance each month?

• Relevant when:
Q64. Zambia has launched the National Health Insurance for Zambian Households to access health services without financial hardship. Do you currently have any health insurance = No.

[Q68_reg]
Q68.As part of this project, we offer all respondents support with enrollment. If you are interested, we can register you on the scheme's website. We will need your personal details, NRC and monthly income. Will you enroll in the scheme if I help you with the registration process or explain how the scheme works?

Choose only one option:

○	Yes
○	No

• Relevant when:
Q68.As part of this project, we offer all respondents support with enrollment. If you are interested, we can register you on the scheme's website. We will need your personal details, NRC and monthly income. Will you enroll in the scheme if I help you with the registration process or explain how the scheme works? = No.

[Q69_reason]
Q69.What is the main reason you will not enroll?

Choose only one option:

○	Cannot afford the premium
○	Do not need insurance
○	Poor quality of care
○	Do not trust government programs
○	Do not understand the scheme
○	None
○	Other

• Relevant when:
Q69.What is the main reason you will not enroll? = Other.

[Q69a__oth]
Q69a.Specify other

• Relevant when:
Q69.What is the main reason you will not enroll? ≠ None || Q68.As part of this project, we offer all respondents support with enrollment. If you are interested, we can register you on the scheme's website. We will need your personal details, NRC and monthly income. Will you enroll in the scheme if I help you with the registration process or explain how the scheme works? = Yes || Q64. Zambia has launched the National Health Insurance for Zambian Households to access health services without financial hardship. Do you currently have any health insurance = Yes.

[Q70_reason]
Q70.Is there another reason you will not enroll?

Choose only one option:

○	Cannot afford the premium
○	Do not need insurance
○	Poor quality of care
○	Do not trust government programs
○	Do not understand the scheme
○	None
○	Other

• Relevant when:
Q70.Is there another reason you will not enroll? = Other.

[Q70a_oth]
Q70a.Specify other

• Relevant when:
Q70.Is there another reason you will not enroll? ≠ None || Q68.As part of this project, we offer all respondents support with enrollment. If you are interested, we can register you on the scheme's website. We will need your personal details, NRC and monthly income. Will you enroll in the scheme if I help you with the registration process or explain how the scheme works? = Yes || Q64. Zambia has launched the National Health Insurance for Zambian Households to access health services without financial hardship. Do you currently have any health insurance = Yes.

[Q71_reason]
Q71.Is there another reason you will not enroll?

Choose only one option:

○	Cannot afford the premium
○	Do not need insurance
○	Poor quality of care
○	Do not trust government programs
○	Do not understand the scheme
○	None
○	Other

• Relevant when:
Q71.Is there another reason you will not enroll? = Other.

[Q71_oth]
Q71a.Specify other

[telephone]
Telephone number please?
